# Diabetes Mellitus as a Risk Factor for Open-Angle Glaucoma: A Systematic Review and Meta-Analysis

**DOI:** 10.1371/journal.pone.0102972

**Published:** 2014-08-19

**Authors:** Minwen Zhou, Wei Wang, Wenbin Huang, Xiulan Zhang

**Affiliations:** 1 Zhongshan Ophthalmic Center, State Key Laboratory of Ophthalmology, Sun Yat-Sen University, Guangzhou, People's Republic of China; 2 Department of Ophthalmology, Shanghai First People's Hospital, School of Medicine, Shanghai JiaoTong University, Shanghai, China; 3 Shanghai Key Laboratory of Fundus Disease, Shanghai, China; Massachusetts Eye & Ear Infirmary, Harvard Medical School, United States of America

## Abstract

**Objective:**

To determine the association between diabetes mellitus (DM) and primary open-angle glaucoma (POAG).

**Methods:**

This is a systematic review and meta-analysis of case-control and cohort studies. The literature search included two databases (PubMed and Embase) and the reference lists of the retrieved studies. Separate meta-analyses for case-control studies and cohort studies were conducted using random-effects models, with results reported as adjusted odds ratios (ORs) and relative risks (RRs), respectively.

**Results:**

Thirteen studies—seven case-control studies and six population-based cohort studies—were included in this meta-analysis. The pooled RR of the association between DM and POAG based on the risk estimates of the six cohort studies was 1.40 (95% CI, 1.25–1.57). The pooled OR of the association between DM and POAG based on the risk estimates of the seven case-control studies was 1.49 (95% CI, 1.17–1.88). There was considerable heterogeneity among the case-control studies that reported an association between DM mellitus and POAG (*P*<0.001) and no significant heterogeneity among the cohort studies (*P* = 0.377). After omitting the case-control study that contributed significantly to the heterogeneity, the pooled OR for the association between DM and POAG was 1.35 (95% CI, 1.06–1.74).

**Conclusions:**

Individuals with DM have an increased risk of developing POAG.

## Introduction

Glaucoma is a significant cause of irreversible blindness worldwide [1]. Primary open angle glaucoma (POAG) is the most common type [2]. Older age [3], a family history of POAG [4], myopia [5], central corneal thickness [6], and ocular hypertension [7], [8] are relatively consistent risk factors for POAG.

Diabetes mellitus (DM) is a serious and increasingly prevalent health problem worldwide due to lifestyle changes and an aging population. DM is associated with severe acute and chronic complications, which negatively influence both the quality of life and the survival of affected individuals [9]. The prevalence of diabetes among all age groups worldwide was estimated to be 2.8% in 2000 and 4.4% in 2030 [10]. Some studies also found that DM is another possible risk factor for POAG [11], [12], [13], [14]. However, the relationship between DM and POAG is controversial [15], [16].

An earlier meta-analysis in 2004 indicated that DM is associated with an increased risk of developing POAG [17]. However, that meta-analysis of seven cross-sectional and five case-control studies of DM and the risk of POAG did not include any cohort studies. The major drawback of cross-sectional studies is that they cannot establish clear temporal relationship between exposure and outcome. Prospective cohort studies would be a good way to assess the relationship between exposure to diabetes and development of POAG. Since then, much larger-scale epidemiological evidence, especially cohort studies, of an association between DM and POAG has been reported [13], [15], [18], [19], [20], [21], [22]. However, these epidemiological studies of the relationship between DM and POAG were somewhat contradictory and inconclusive, and two studies reported discordant results [15], [23].

To provide a more accurate estimate of the association between DM and POAG, we conducted a meta-analysis of all case-control and cohort studies involving DM and POAG.

## Methods

### Search strategy

We conducted a systematic review and meta-analysis to examine the association of DM with POAG based on the Meta-analysis of Observational Studies in Epidemiology guidelines [24]. We conducted a computerized search of the PubMed and Embase up to Feb 08, 2014. The following terms, adapted for each database, were used for the searches: diabetes mellitus, DM, impaired glucose tolerance, hyperglycemia, insulin resistance, insulin secrete dysfunction, glaucoma, intraocular hypertension, intraocular pressure, ocular hypertension. To achieve maximum sensitivity, limits or filters were not placed on the searches. No language restrictions were imposed. A manual search was performed by checking the reference lists of the original reports.

### Inclusion and exclusion criteria

The full-length articles were required to meet the following inclusion and exclusion criteria for the meta-analysis. The inclusion criteria were: (1) they had a case-control or a cohort design, (2) the exposure of interest was DM, (3) the outcome of interest was POAG, and (4) the odds ratios (ORs) or the relative risk (RR) estimates with their 95% confidence intervals (CIs) (or data to calculate them: raw data, *P* value, and/or variance estimates) were reported.

We excluded the following: (1) studies involving secondary glaucoma or angle-closure glaucoma, (2) studies without a detailed description of the POAG assessment, (3) crude data that could not calculate the adjusted ORs or the adjusted RRs. When multiple publications from the same study population were available, we checked for duplicate analyses and included only the most recent publication. We excluded one study that reported only crude data because the ORs and their 95% CIs could not be calculated.

### Data extraction and quality assessment

Two authors (Z.M.W. and W.W.) independently extracted the following data from each publication: publication data (author, year of publication, and country of the population studied); study design (cohort study or case-control study); methods of DM ascertainment (self-report, medical records, and blood glucose measurement); definition of glaucoma; type of DM; participant's age; study population; number of cases and controls (for case-control studies); number of exposed and comparison group (for cohort studies); number of cases (for cohort studies); number of DM patients (for case-control studies); follow-up period (for cohort studies); summary estimates and corresponding 95% CI, and confounding factors adjusted for.

Two reviewers independently assessed the quality of each study using the Newcastle-Ottawa Scale (NOS) [25]. The NOS consists of three parameters of quality: selection, comparability, and exposure (case-control studies) or outcome (cohort studies). The NOS assigns a maximum of four points for selection, two points for comparability, and three points for exposure/outcome. Therefore, nine points reflect the highest quality. Any discrepancies were addressed by a joint re-evaluation of the original article with a third reviewer.

### Statistical analyses

The data from the cohort studies and the case-control studies were analyzed separately. The RR was used as a common measure of the association between DM and the risk of POAG in the cohort studies. The incidence rate ratio (IRR) and the hazard ratio (HR) were considered as RRs, and the pooled adjusted RRs with the corresponding 95% CIs were calculated. For the case-control studies, the pooled adjusted ORs with the corresponding 95% CIs were calculated. The maximally adjusted RRs or ORs were used to assess the association between DM and POAG. Considering the differences in the characteristics of the study groups and the variation in the sample sizes, we assumed that heterogeneity was present even when no statistical significance was identified. Thus, we combined the data using a random-effects model. Statistical heterogeneity between the studies was evaluated using Cochran's Q test and the I^2^ statistic. For the Q statistic, *P*<0.05 was considered to indicate statistically significant heterogeneity. For the case-control studies, a sensitivity analysis was also conducted, in which one study at a time was removed and the rest were analyzed to estimate whether a single study could have markedly affected the results. For the cohort studies, stratified analysis was performed according to the type of DM, type of effect measure, and geographic area. To detect publication biases, we calculated Begg and Egger measures. *P*<0.05 was considered statistically significant in the test for overall effect. The analysis was conducted using the Stata software package (Version 11.0; Stata Corp., College Station, TX).

## Results

### Identification and selection of reports for the systematic review and meta-analysis

**Figure 1:**
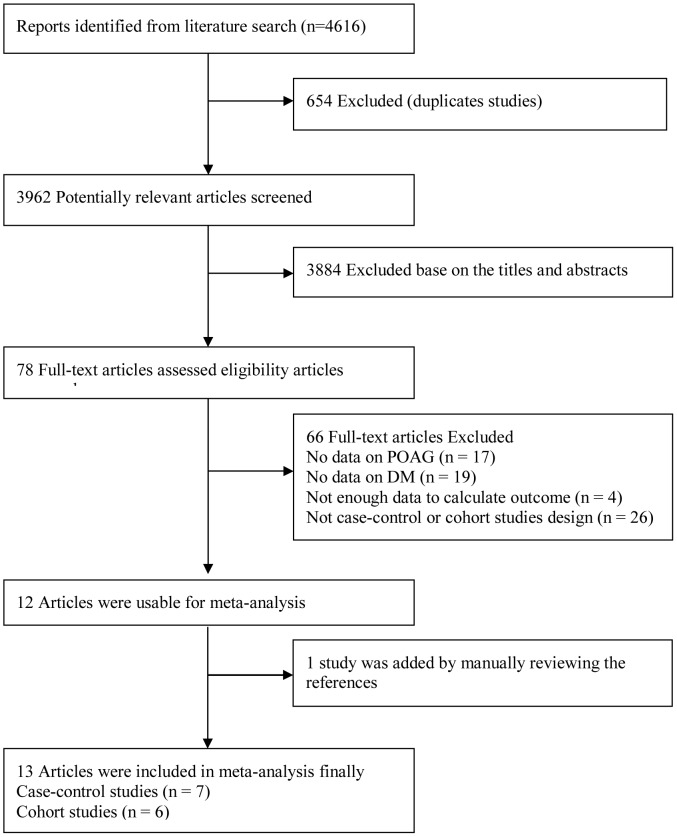
Flow diagram outlining the selection process for the inclusion of the studies in the systematic review and meta-analysis. POAG = primary open-angle glaucoma; DM = diabetes mellitus.

We identified 4616 articles from the database search. After the removal of 654 duplicate publications, there were 3962 studies. In total, 78 articles were retrieved for full-text review. Finally, we identified seven case-control studies [21], [22], [26], [27], [28], [29], [30] and six cohort studies [13], [15], [18], [19], [20], [23] that presented results on DM and the risk of POAG (Figure 1). One cohort study was excluded because it only provided an unadjusted but not adjusted RR [31].

### Characteristics of the case-control studies

The main characteristics of the case-control studies are presented in Table 1. The studies were published between 1987 and 2009. Three studies originated from the United States, one from Korea, one from the Congo, and two from Europe (France and Denmark). In total, 11,472 cases and 75,631 controls were included in this meta-analysis. Four studies reported a positive association between DM and the incidence of POAG. The definition of POAG varied across the studies. Four studies included an increased IOP in their case definition of POAG [26], [27], [28], [30], and one study included a history of glaucoma treatment [21]. Six studies included disc cupping abnormalities (a measure of optic nerve damage) and the visual field test [22], [26], [27], [28], [29], [30]. DM was ascertained by self-reporting [26], [27], [29], [30], medical records [21], [22], and blood glucose level [28].

**Table 1:** Characteristics of case-control studies of DM and POAG incidence.

Author (year)	Location	Definition of glaucoma	DM type	Study population	POAG patients	control subjects	Age (case/control)	DM patients (case/control)	Adjusted OR (95%CI)	Adjusted Covariates
Welinder (2009) [21]	Denmark	GON, GVFL	Type 1 and 2	Population-based	5,991	59,910	60.8–78.4/60.8–78.4	704/3975	1.81 (1.65–1.98)	1,2,4,17–20
Motsko (2008) [22]	United States	GON, GVFL	NA	Population-based	4728	14184	73.6±7.6/73.5±7.6	1333/3126	1.33 (1.23–1.44)	1,2
Kaimbo (2001) [28]	Congo	GON, GVFL	Type 1 and 2	Hospital-based	40	104	28–80/31–81	5/4	4.2 (0.74–22.2)	1,3,5
Charliat (1994) [29]	France	GON, GVFL	NA	Hospital-based	175	175	62.8±10.1/62.8±10.0	12/20	0.61 (0.28–1.30)	1,2
Uhm (1992) [27]	Korea	GON, GVFL	NA	Hospital-based	361	927	66.89±13.13/54.66±18.21	71/138	1.40 (1.02–1.92)	1,3,4,9
Katz (1988) [30]	United States	GON, GVFL	NA	Hospital-based	94	94	59±3/59±3	14/5	2.80 (1.01–7.77)	1–3
Wilson (1987) [26]	United States	GON, GVFL	NA	Hospital-based	83	237	NA	10/16	1.6(0.5–5.2)	1–4,8,9,15,16

1, age; 2, gender;3, race; 4, Hypertension; 5, body mass index; 6, physical activity; 7, alcohol intake; 8, Smoking; 9, family history of glaucoma; 10, follow-up time; 11, IOP; 12, IOP–lowering treatment; 13, questionnaire cycle; 14, education; 15, Myopia; 16, Radiation exposure; 17, thyroid disease; 18, migraine; 19, cardiovascular events; 20, angiotensin-converting enzyme inhibitors.

DM: diabetes mellitus; OR:odds ratio; CI: confidence Interval; GVFL: Glaucomatous visual field loss; GON: Glaucomatous optic neuropathy.

### Characteristics of the cohort studies

The main characteristics of the cohort studies are listed in Table 2. The studies were published between 2000 and 2011. Three studies originated from the United States, one from the Netherlands, one from the Barbados, and one from the United Kingdom. A total of 46,360 cases of POAG in a cohort of 3,393,011 individuals were included in this meta-analysis. Three studies reported a positive association between DM and POAG. The definition of POAG varied across the studies. Two studies included an increased intraocular pressure (IOP) in their case definition of POAG [13], [18], and all the studies included a visual field test and disc cupping abnormalities [13], [15], [18], [19], [20], [23]. DM was ascertained by self-reporting [13], [18], [23], medical records [20], and the patient's blood glucose level [15], [19].

**Table 2:** Characteristics of cohort studies of DM and POAG incidence.

Author (year)	Location	Definition of glaucoma	DM type	Study population	Exposed group	Comparison group	Age	Case (EG/CG)	Adjusted RR (95%CI)	Adjusted Covariates	Follow-up(year)
Wise (2011) [18]	United States	GON, GVFL	Type 2	32,570 Population-based	1,055	31,294	21–69	57/308	1.58 (1.17– 2.13)*	1,4–8,12,13	12
Newman-Casey (2011) [19]	United States	GON, GVFL	Type 1 and 2	2,182,315 Population-based	22,500	2,159,815	54.5±10.3	506/43825	1.35 (1.21–1.50)#	1–5	6
Leske (2008) [23]	Barbados	GON, GVFL	NA	3222 Population-based	536	2675	56.9±11.3	NA	1.2 (0.7–1.8)	1,2	9
de Voogd (2006) [15]	Netherland	GON, GVFL	Type 1 and 2	3837 Population-based	264	3573	65.7±6.9	5/82	0.65 (0.2–1.64)	1,2,4,5,10, 11	6.5
Pasquale (2006) [13]	United States	GON, GVFL	Type 2	998,292 Population-based	32,362	965,930	≥40	30/399	1.82 (1.2–2.70)	1–9	20
Ellis (2000) [20]	United Kingdom	GON, GVFL	Type 1 and 2	172,775 Population-based	6631	166,144	>40	65/958	1.57 (0.99–2.48)	1	2

1, age; 2, gender; 3, race; 4, Hypertension; 5, body mass index; 6, physical activity; 7, alcohol intake; 8, Smoking; 9, family history of glaucoma; 10, follow-up time; 11, IOP; 12, questionnaire cycle; 13, education.

DM: diabetes mellitus; EG: exposed group; CG: comparison group; RR: risk ratio; CI: confidence Interval; GVFL: Glaucomatous visual field loss; GON: Glaucomatous optic neuropathy;

*: Incidence rate ratio;

# : hazard ratio.

### Quality assessment results

With regard to the case-control studies, all the studies were of high quality (NOS score >6), with an average NOS score of 7.43. All the cohort studies were also of high quality (NOS score >6), with an average NOS score of 7.83. The most common bias was ascertainment of exposure, with four studies assessing DM by self-reporting or medical records.

### Pooled estimates of the association between DM and POAG analysis with the case-control studies

**Figure 2:**
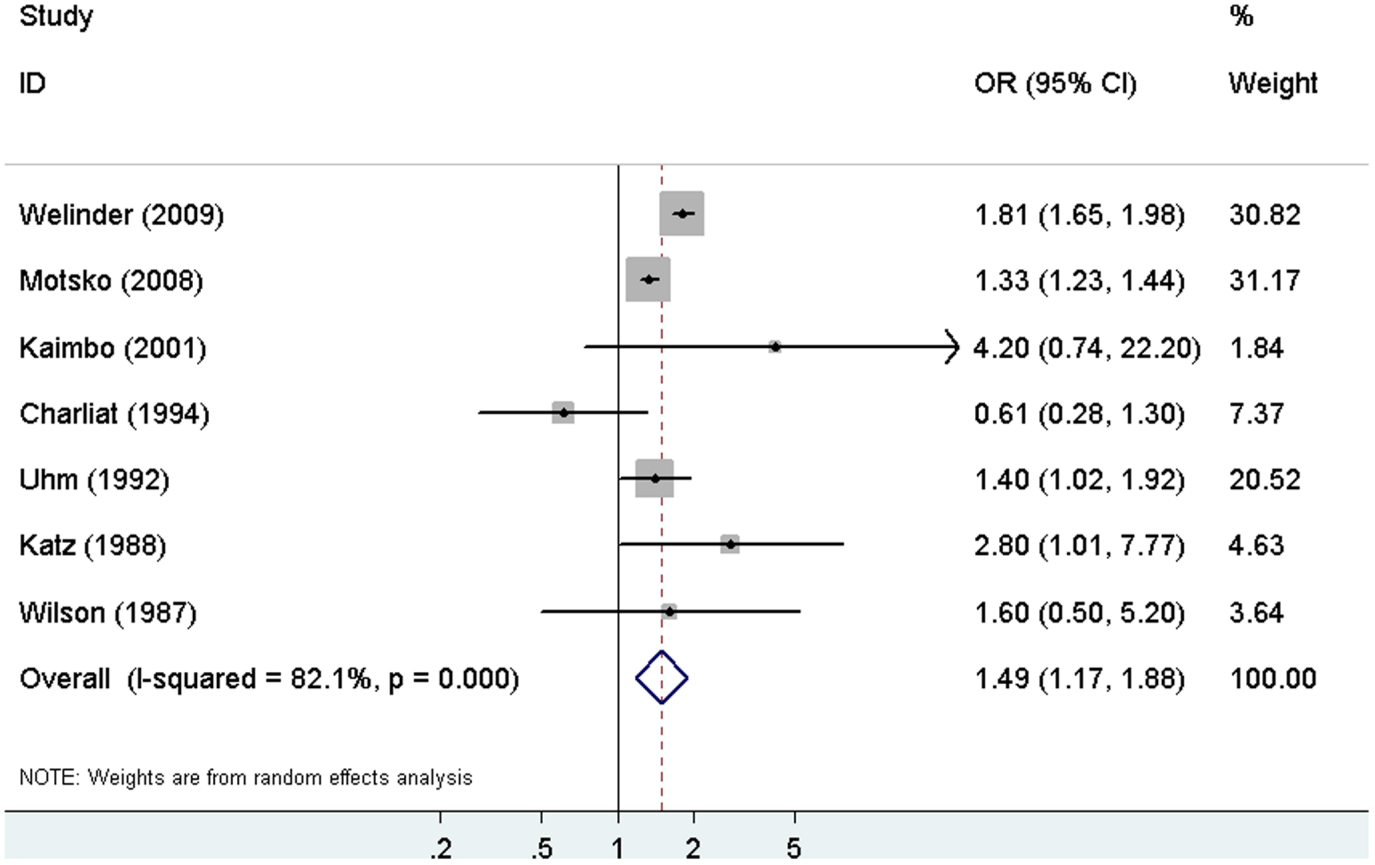
Forest plot of the risk estimates of the association between DM and POAG in the case-control studies. OR = odds ratio; CI = confidence interval; DM = diabetes mellitus; POAG = primary open-angle glaucoma.

The pooled OR for the seven case-control studies was 1.49 (95% CI, 1.17–1.88) under the random-effects model. Figure 2 shows the multivariate ORs for each study separately and for the seven case-control studies combined. There was statistically significant heterogeneity among the seven case-control studies (I^2^ = 82.1%; *P*<0.001). The sensitivity analysis showed that Welinder's study [21] substantially influenced the pooled OR (Table 3). After excluding this study, the pooled OR was 1.35 (95% CI, 1.06–1.74), with no evidence of heterogeneity (I^2^ = 37.1%; *P* = 0.159).

**Table 3:** Sensitivity analysis of case-control study.

	Random Effects Model		Test of Homogeneity		
Study Excluded	OR	95%CI	Q	I^2^ (%)	*P*-value
None	1.49	1.17, 1.88	33.53	82.1	<0.001
Charliat (1994)	1.59	1.26, 2.00	28.14	82.2	<0.001
Uhm (1992)	1.51	1.14, 1.99	33.30	85.0	<0.001
Kaimbo (2001)	1.46	1.15, 1.85	32.13	84.4	<0.001
Welinder (2009)	1.35	1.06, 1.74	7.96	37.1	0.159
Wilson (1987)	1.48	1.16, 1.89	33.52	85.1	<0.001
Motsko (2008)	1.55	1.12, 2.14	11.52	56.6	0.042
Katz (1988)	1.44	1.13, 1.84	32.11	84.4	<0.001

OR: odds ratio; CI: confidence Interval.

### Analysis with the cohort studies

**Figure 3:**
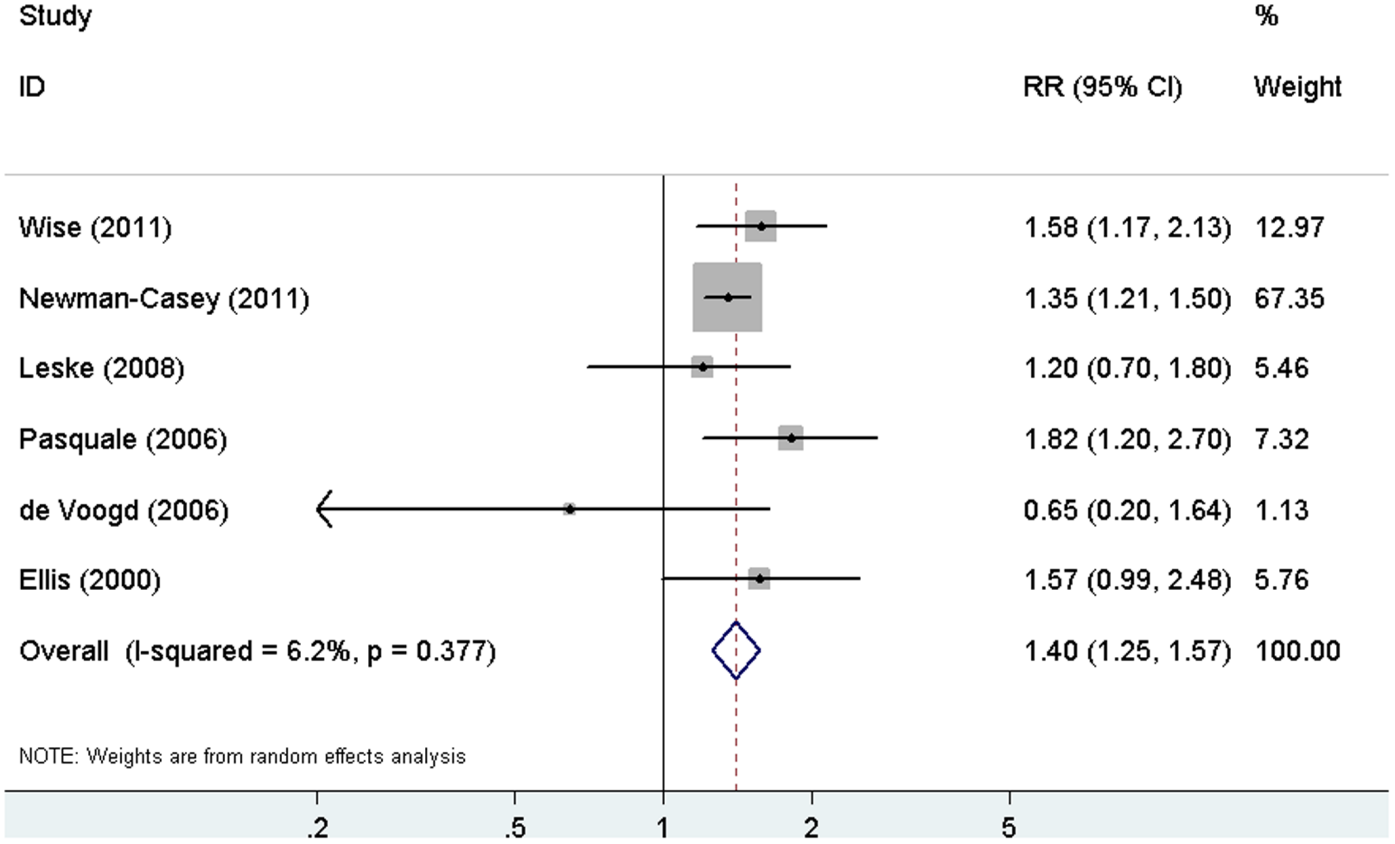
Forest plot of the risk estimates of the association between DM and POAG in the cohort studies. RR = risk ratio; CI = confidence interval; DM = diabetes mellitus; POAG = primary open-angle glaucoma.

The pooled RR for the five cohort studies was 1.40 (95% CI, 1.25–1.57) under the random-effects model, and the heterogeneity was statistically insignificant (I^2^ = 6.2%; *P* = 0.377). Figure 3 shows the multivariate RRs for each study separately and for the six cohort studies combined. Subgroup analyses according to type of DM, type of effect measure, and geographic area were performed to examine the impacts of these factors on the association (Table 4). No evidence of heterogeneity was observed within any subgroup. A significant positive association between DM and POAG was observed in all subgroups.

**Table 4:** Subgroup Meta-analyses of cohort study.

		Random Effects Model		Overall Effect		Test of Homogeneity		
Subgroup	No. Studies	RR	95%CI	Z	*P*	Q	I^2^ (%)	*P*
Type of DM								
DM type 1 and 2	3	1.35	1.13, 1.61	3.34	0.001	2.27	11.9	0.322
DM type 2	2	1.66	1.31, 2.11	4.13	<0.001	0.30	0.0	0.582
Type of effect measure								
RR value	4	1.43	1.06, 1.94	2.31	0.021	4.17	28.0	0.244
Non-RR value	2	1.37	1.24, 1.52	6.16	<0.001	0.94	0.0	0.333
Geographic area								
United States	3	1.38	1.14, 1.67	3.31	0.001	2.87	30.2	0.238
Other countries	3	1.54	1.19, 1.99	3.31	0.001	1.73	0.0	0.421

RR: risk ratio; CI: confidence Interval; DM: diabetes mellitus.

### Publication bias

**Figure 4:**
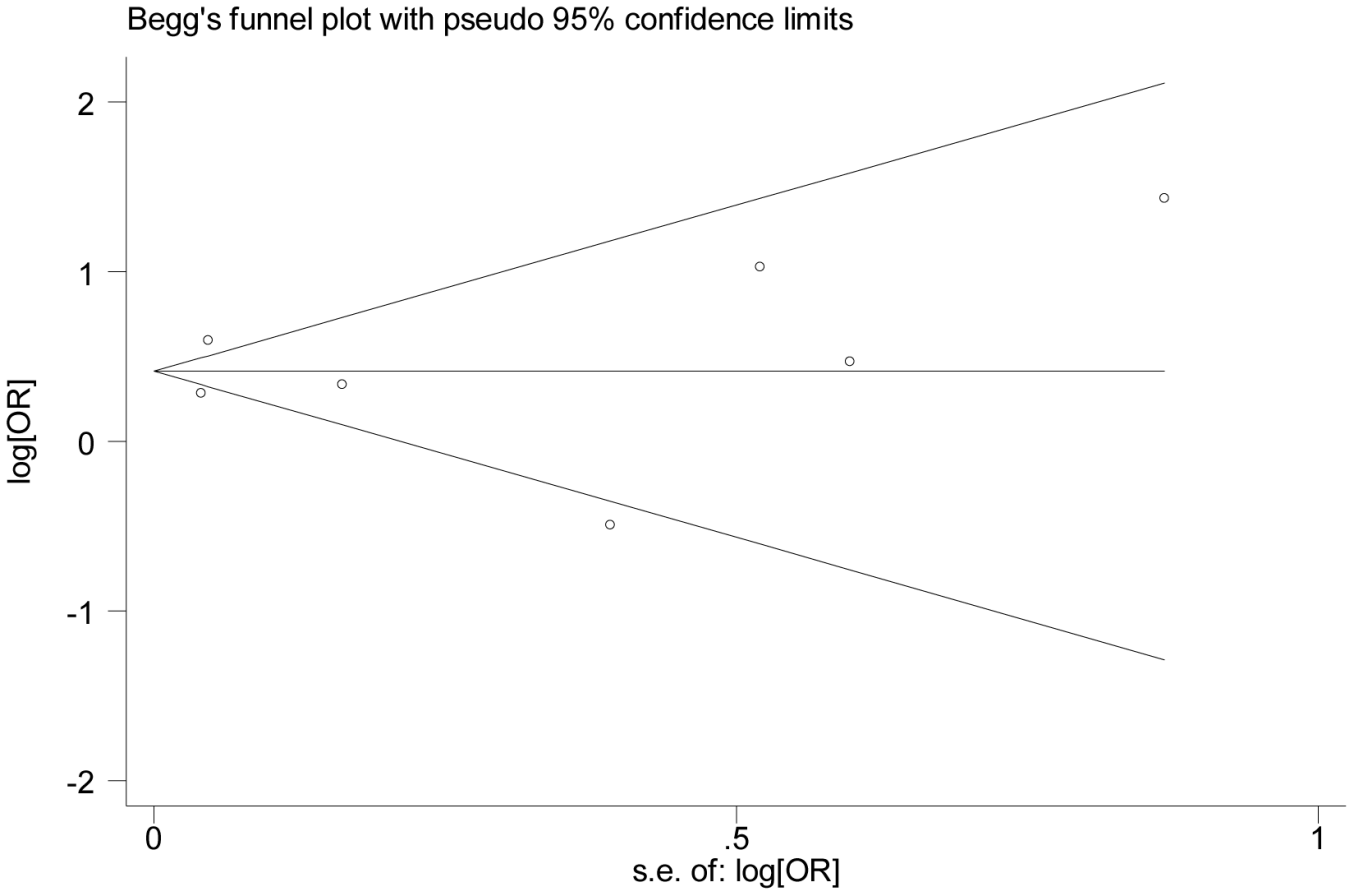
Funnel plot of the case-control studies evaluating the association between DM and POAG. Begg's regression asymmetry test (*P* = 0.548). OR = odds ratio; DM = diabetes mellitus; POAG = primary open-angle glaucoma.

**Figure 5:**
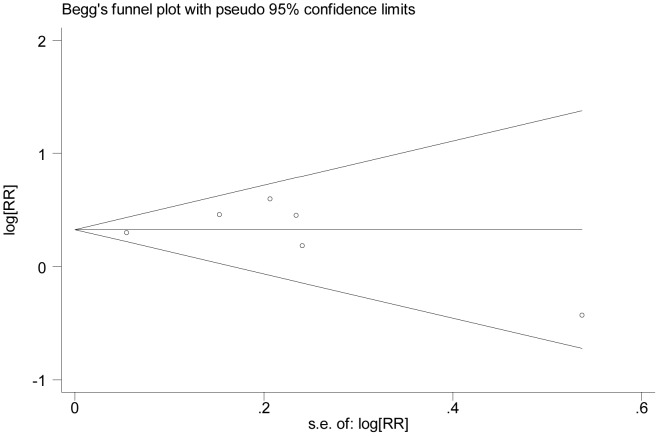
Funnel plot of the cohort studies evaluating the association between DM and POAG. Begg's regression asymmetry test (*P* = 0.452). RR = risk ratio; DM = diabetes mellitus; POAG = primary open-angle glaucoma.

The assessment of publication bias (Figure 4) using Begg's test (*P* = 0.548) and Egger's test (*P* = 0.939) doesn't show evidence of publication bias in the case-control studies. Publication bias also was not statistically detected by Begg's test (*P* = 0.452) and Egger's test (*P* = 0.923) in the cohort studies (Figure 5).

## Discussion

Although several risk factors for the development of POAG have been evaluated, this is a field of ongoing investigation [32]. DM is a possible risk factor for POAG. The current literature does not provide a definitive link between DM and POAG. Hence, the purpose of this systematic review and meta-analysis was to summarize all the available relevant evidence with reference to the relationship between DM and POAG. The findings from this meta-analysis support the evidence for a positive relation between DM and an increased risk of POAG. The results, based on 13 epidemiological studies, are consistent with those of a previous meta-analysis [17]. However, unlike previous studies, we included several newly published case-control and cohort studies, and we excluded cross-sectional studies in this updated meta-analysis. This allowed for a greater number of subjects and, hence, a more detailed and accurate risk estimation than in prior meta-analyses.

The results from the case-control and cohort studies were quite similar. The findings from this meta-analysis showed that compared with non-diabetic individuals, individuals with DM have an approximately 1.4-fold increased risk of developing POAG in cohort studies. The results from the case-control studies showed that they have an about 49% increased odds of developing POAG compared with individuals without DM. Moreover, the results from the cohort studies subgroup analyses were quite similar, with a significant association found between DM and POAG in all the subgroups. The results were not substantially affected by the DM type. In the current meta-analysis, no study was excluded based on sample size, and both Begg's test and Egger's test doesn't show evidence of publication bias. Thus, the results of this meta-analysis are robust.

Heterogeneity is often a concern in a meta-analysis. Substantial heterogeneity was observed in case-control studies, which was expected given the between-study variation, such as inconsistent data collecting methods, different ethnic populations, and different sample size. In the leave-one-out sensitivity procedure, we found that removing Welinder's study [21] from the case-control studies altered the results. This study had the largest sample size and the greatest differences in the strength of the association. These factors may have been the main sources of the heterogeneity. After this study was excluded, the remainder showed a positive association between DM and POAG, with no evidence of heterogeneity. However, the high quality of Welinder's study showed the positive relation between DM and POAG, which is consist with the pooled outcome. Thus, despite exist the heterogeneity in case-control studies, the results of this meta-analysis are still robust. Little evidence of heterogeneity was observed in the subgroups of the cohort studies.

There seems to be a direct relationship between DM and POAG. Several hypotheses on biological links between DM and POAG have been proposed. First, there is a growing body of evidence that the presence of long-standing hyperglycemia, along with lipid anomalies, may increase the risk of neuronal injury from stress [33]. In particular, laboratory data have provided robust evidence for such an association [34], [35]. Second, studies showed that diabetic eyes have a reduced capacity to auto-regulate blood flow and that they exhibit decreased retinal blood flow [36]. As a result, they show relative hypoxia and overexpression of hypoxia-inducible factor-1 (HIF-1α [37], [38]. Importantly, levels of HIF-1α increased in ganglion cells, in the retina, and in the optic nerve head of human glaucomatous eyes in response to elevated IOP [39]. These might be another important association between DM and POAG. Third, the observed association between DM and POAG may be explained by the remodeling of the connective tissue of the optic nerve head. The remodeling might reduce compliance at the trabecular meshwork and the lamina cribrosa, resulting in increased IOP and greater mechanical stress on the optic nerve head, respectively [40], [41]. Research has demonstrated that diabetes can exacerbate connective tissue remodeling and amplify these biomechanical changes [42]. More importantly, the Barbados Eye Study had found that diabetes was a risk factor for increased IOP in follow-up [43]. Genetic factors and diabetes-related autonomic dysfunction are likely to play a role of this association [44]. Further research is needed to better understand these phenomena.

The strengths of the present study are as follows: First, our meta-analysis of 13 studies involving a relatively large number of cases and participants enhanced the power to detect a significant association and provided more reliable estimates. Second, most of the included studies evaluated multiple potential confounding factors, some of which were considered to be risk factors for POAG, such as race, hypertension, family history of glaucoma, smoking, and body mass index. Third, the large majority of the studies included were of high quality.

As with any meta-analysis of observational studies, there are several potential limitations with regard to the results. First, significant heterogeneity existed in the case-control studies. We found that one study was the main source of this heterogeneity, but when we exclude this study, the remainder studies still showed the similar association between DM and POAG. Second, in the cohort studies, the effect size of one study was the IRR; it was the HR in another study but not the RR effect size. However, the positive association between DM and POAG was consistently observed in RRs and non-RRs subgroup. Third, DM was self-reported in some studies, and this may have introduced a recall bias. Fourth, in general, publication bias is a major problem in published studies and in meta-analyses of published studies. Publication bias may be an issue because studies that report statistically significant results are more likely to be published than studies that report non-significant results, and this could have distorted the findings of our meta-analyses [45]. However, the results obtained from the funnel plot analysis and the formal statistical tests did not provide evidence for such bias. Finally, no cohort studies addresses detection bias and it is another limitation in this meta-analysis.

Our findings have important public health implications. Glaucoma is an important cause of irreversible blindness worldwide. Controversy continues regarding the effects of DM on the risk of POAG. The findings from our study aimed at addressing this issue and resolving the inconsistency are both important and timely. In summary, the results of this meta-analysis point to a significant association between DM and the risk of POAG. Further studies are needed to elucidate the exact underlying mechanisms linking DM with POAG.

## Supporting Information

Checklist S1 **PRISMA Checklist.**
(DOC)
